# A Retrospective Review of Microbiological Methods Applied in Studies Following the Deepwater Horizon Oil Spill

**DOI:** 10.3389/fmicb.2018.00520

**Published:** 2018-03-23

**Authors:** Shuangfei Zhang, Zhong Hu, Hui Wang

**Affiliations:** Biology Department, College of Science, Shantou University, Shantou, China

**Keywords:** pure culture, clone library, denaturing gradient gel electrophoresis, pyrosequencing, meta-omics, stable isotope probing, molecular ecological networks

## Abstract

The Deepwater Horizon (DWH) oil spill in the Gulf of Mexico in 2010 resulted in serious damage to local marine and coastal environments. In addition to the physical removal and chemical dispersion of spilled oil, biodegradation by indigenous microorganisms was regarded as the most effective way for cleaning up residual oil. Different microbiological methods were applied to investigate the changes and responses of bacterial communities after the DWH oil spills. By summarizing and analyzing these microbiological methods, giving recommendations and proposing some methods that have not been used, this review aims to provide constructive guidelines for microbiological studies after environmental disasters, especially those involving organic pollutants.

## The role of microbes during the DWH oil spill

The DWH oil rig exploded in the Gulf of Mexico in April 2010. The spill lasted for approximately 84 d, which was recorded the largest oil spill in United States to date (Crone and Tolstoy, 2010). The accident yielded an unremitting flow of oil that ultimately totaled 4.9 million barrels (779 million liters, ±10%) (Lehr et al., 2010). An oil plume that stretched for more than 35 km was observed (Camilli et al., 2010; Hazen et al., 2010). Crude oils are composed of thousands of chemicals, which mainly include saturated hydrocarbons, aromatic hydrocarbons, and the more polar, non-hydrocarbon components in resins and asphaltenes (Head et al., 2006). These four main components have different levels of toxicity to marine organisms and vary in proportion with different types of crude oil. Exposure to large amounts of oil in marine environments can cause significant negative impacts on resident organisms and human health (Grattan et al., 2011). Oil spills can lead to acute and chronic environmental damage, and they can have toxic effects on the aquatic ecosystems (Campagna et al., 2011; Hu et al., 2011; Whitehead et al., 2011).

By 20 August 2010, 78% of the oil from the DWH oil spill was disposed by either human intervention (direct recovery, *in situ* burning, skimming, and dispersal) or natural processes (naturally dispersed, evaporated, and dissolved) (Ramseur, 2010), whereas the fate of the remaining 22% of the oil spill was uncertain. Microorganisms, especially bacteria, are believed to act as the key players in bioremediation of residual oil (Head et al., 2006; Wang et al., 2013). The ability to degrade hydrocarbon components of crude oil has been widely detected among different bacteria (Head et al., 2006). Considering the large amount of oil released from the DWH oil spill and specific features of the rig (the broken riser pipe was at a depth of 1,500 m below the surface), the responses and roles of bacteria following the spill have drawn significant attention from scientists (Kimes et al., 2014). Results from different research groups indicated that indigenous bacterial communities in different habitats (seawater, deep-sea sediment, marshes, and beach sands) responded rapidly to the spilled oil and that some bacterial groups might play significant roles in reducing the environmental contamination (Table 1). Moreover, sunlight and temperature were found to be the key factors which influenced the composition of bacterial communities and selected for oil-degrading bacteria in surface water and bottom water, respectively (Bacosa et al., 2015b; Liu et al., 2017).

**Table 1:** Responses of bacterial communities to the Deepwater Horizon oil spill and various microbiological methods applied in different studies.

**Habits**	**Sampling time**	**Elapsed time (months)**	**Dominant bacterial phylotypes**	**Technologies**	**References**
Sediments	09/2010–10/2010	5–6	Uncultured γ-*proteobacteria* and *Colwellia*	16S rRNA gene-based clone library, metagenomics, and SIP	Mason et al., 2014b
	12/2010	8	Uncultured γ-*proteobacteria* and δ-*Proteobacteria* (*Desulfobacterales, Desulfuromonadales*, and *Desulfarculales*)	16S rRNA gene-based clone library, and Illumina amplicon sequencing	Simister et al., 2016
	11/2011	19	*Oceanospirillales* and *Colwellia*	Metagenomics and metatranscriptomics	Yergeau et al., 2015
Deep water		Before spill	α-*proteobacteria, Pelagibacter* and *Actinobacteria*	PhyloChip, 16S rRNA gene-based clone library, and SIP	Hazen et al., 2010
	05/2010	1	γ-*proteobacteria* (*Oceanospirillales, Alteromonadales, Cycloclasticus*,	454 pyrosequencing	Joye et al., 2014
	05/2010 06/2010	1–2	γ-*proteobacteria* (*Oceanospirillales, Oleispira, Spongiispira, Cycloclasticus, Oleiphilus, Thalassolituus*, and *Colwellia*)	PhyloChip, 16S rRNA gene-based clone library, and SIP	Hazen et al., 2010; Valentine et al., 2010; Redmond and Valentine, 2011; King et al., 2015
	09/2010	5	Methylotrophic bacteria (*Methylococcaceae, Methylophaga*, and *Methylophilaceae*)	16S rRNA gene-based clone library	Kessler et al., 2011
	11/2011	19	γ-*proteobacteria*	Metagenomics and metatranscriptomics	Yergeau et al., 2015
Surface water		Before spill	α-*proteobacteria, Pelagibacter*, and *Actinobacteria*.	PhyloChip, 16S rRNA gene-based clone library, and SIP	Hazen et al., 2010
	05/2010	1	*Oceanospirillales* and *Rhodospirillales*	Metagenomics and SIP	Dombrowski et al., 2016
	05/2010	1	*Alcanivorax, Marinobacter, Alteromonas, Cycloclasticus*, and *Colwellia)-*	SIP	Gutierrez et al., 2013
	06/2010	2	*Cyanobacteria* and α-*proteobacteria* (SAR11 clade, *Rhodobacterales*, and *Rhodospirillales*)	16S rRNA gene-based clone library	Chakraborty et al., 2012
	09/2010	5	γ-*proteobacteria* (*Pseudoalteromonas, Pseudomonas, Vibrio, Acinetobacter*, and *Alteromonas*)	PhyloChip, 16S rRNA gene-based clone library, and SIP	Redmond and Valentine, 2011
	11/2011	19	*Cyanobacteria*	Metagenomics and metatranscriptomics	Yergeau et al., 2015
Marsh sediments	06/2010, 07/2010, and 09/2010	2–5	*Proteobacteria, Bacteroidetes, Actinobacteria*, and *Firmicutes* (*Bacilli* and *Clostridia*)	PhyloChip and GeoChip	Beazley et al., 2012
	05/2010, 09/2010, 06/2011, 09/2011, and 08/2013	1–40	*Proteobacteria, Firmicutes, Bacteroidetes*, and/or *Chloroflexi* phyla	GS FLX amplicon pyrosequencing	Engel et al., 2017
	The fall of 2011, the spring of 2012, and the fall of 2013	18–36	*Desulfococcus, Marinobacter*, and *Mycobacterium*	Metagenomics	Atlas et al., 2015
	07/2012	24	*Nitrosopumilus* cluster	TRFLP Analysis	Bernhard et al., 2016
Beach sands	07/2010	3	α-*proteobacteria* (*Rhodobacteraceae*) and γ-*proteobacteria* (*Alcanivorax, Marinobacter, Pseudomonas*, and *Acinetobacter*)	Pure culture, automated ribosomal intergenic spacer analysis (ARISA), microarray, and SSU rRNA pyro sequence libraries	Kostka et al., 2011
	06/2011	Over 12	α-*proteobacteria* (*Hyphomonas, Parvibaculum*, and *Micavibrio*) and γ-*proteobacteria* (*Alcanivorax, Pseudomonas*, and *Marinobacter*)	Metagenomics and 16S rRNA gene-based clone libraries	Rodriguez-R et al., 2015

The DWH oil spill provides a unique opportunity to understand the significant roles that microbes play in the recovery process at oil-polluted environments and to compare different methods for microbial ecological research. In this review, we focus on analyzing and summarizing various methods applied in investigating the responses of microbial communities to the DWH oil spill. These include both culture-dependent methods (i.e., mainly pure culture) and culture-independent methods [i.e., classical molecular biological methods and several advanced methods, including stable isotope probing (SIP), microarray, metagenomics, metatranscriptomics, and single-cell sequencing]. Additionally, we propose some prevalent methods [e.g., microbial ecosystems metabolic networks (MEMNs) and microbial molecular ecological networks] that exhibit great potential for the future studies.

## Methods for microbiological analysis

### Cultivation-dependent methods

Traditional methods, such as isolation and purification of bacterial strains from various environments are essential as exemplified in microbiological studies of the DWH spill. The isolation of microbes in pure culture can provide valuable information on the functions, metabolic pathways, and physiological characteristics of bacteria that respond to an oil spill. During the DWH spill, different bacterial strains (e.g., *Alcanivorax, Alteromonas, Cycloclasticus, Halomonas, Marinobacter*, and *Pseudoalteromonas*) with hydrocarbon-degrading abilities were isolated from surface slicks and deep oil plumes (Gutierrez et al., 2013). With the addition of oil (or the oil dispersant Corexit) as the sole carbon source, Bælum et al. (2012) successfully isolated one hydrocarbon-degrading strain (belonging to the family *Colwelliaceae*), which were the dominant microorganism in the oil plume.

Despite the successful isolation of some oil-degrading bacteria, most of the indigenous bacterial strains could not be isolated by conventional culture-based methods. This might be attributed to the fact that artificial culture conditions are different from the *in situ* environment, due to the absence of symbiotic organisms and specific nutrient levels (Zengler et al., 2002). Furthermore, specific environmental conditions in deep sea (low temperature, O_2_ restriction and hydrostatic pressure) are always neglected or difficult to setup in the *ex situ* experiments, which limit the isolation of oil-degrading bacteria (Scoma et al., 2016). Recently, some previously uncultured microorganisms were successively grown in pure culture using newly-developed culturing techniques, including (1) supplying various chemical compounds found in their natural environment (Kaeberlein et al., 2002; Rappé et al., 2002; Zengler et al., 2002); (2) lengthening the period of incubation and protecting cells from exogenous peroxides (Stevenson et al., 2004); (3) isolating uncultived microorganisms in a simulated natural environment with specific devices (Kaeberlein et al., 2002); (4) high-throughput dilution to extinction culturing technology (HTC) (Rappé et al., 2002; Stingl et al., 2007). The above microbiological cultivating technologies have not been used to isolate bacteria from the DWH oil spill-contaminated environment, and could be considered in microbiological investigations of future oil spills. Considering the particularity of the deep-sea environment, the method based on the simulated/real natural environment (Kaeberlein et al., 2002) can be applied in future studies. Briefly, surface sediments with *in situ* microorganism can be mixed with warmed agar and sealed in a diffusion chamber sealed by two membranes which not only allow exchange of chemicals between the chamber and the environment, but also restrict the movement of cells. The sealed chambers can be cultivated in a marine aquarium with simulated natural environment (low temperature, O_2_ restriction and hydrostatic pressure) or *in situ* in the deep-sea environment. The development of novel cultivating technologies to isolate previously uncultured bacteria could permit a more comprehensive understanding of unexplored bacterial communities, including their metabolic pathways, and functions.

### Classical molecular biological methods

The majority (>99%) of bacterioplankton in marine environments remains largely recalcitrant to cultivation. Applications of molecular biological methods such as 16S rRNA clone library, terminal restriction fragment length polymorphism (T-RFLP), denaturing gradient gel electrophoresis (DGGE), temperature gradient gel electrophoresis (TGGE), fluorescence *in situ* hybridization (FISH), and real-time quantitative polymerase chain reaction (RT-PCR) are useful techniques in investigating the composition and potential functions of indigenous bacterial communities in a specific environment.

#### 16S rRNA gene clone library

The 16S rRNA gene is well-known as a marker gene for the reconstruction of prokaryotic (bacterial and archaeal) phylogenies and for the investigation of microbial diversity (Woese and Fox, 1977; Klindworth et al., 2013). The construction of clone libraries based on 16S rRNA genes has extensively been used to investigate bacterial communities until the advent of next generation sequencing (NGS). 16S rRNA gene-based clone library analysis provides a useful, though somewhat limited, tool for capturing the diversity and composition of bacterial communities (Kumar et al., 2007; Schäfer et al., 2010). In the literature on the DWH oil spill, 16S rRNA gene-based clone library analysis was one of the most popular techniques for investigating microbial communities. Several studies using 16S rRNA gene clone libraries revealed the microbial community responses to the contamination caused by the deep-sea plumes of hydrocarbons. These communities exhibited significant differences in different habitats (plume and non-plume water, surface and deep-sea water, sediments, and beach sands; see Table 1) and presented dramatic changes over the duration of the spill (Hazen et al., 2010; Valentine et al., 2010; Kessler et al., 2011; Redmond and Valentine, 2011; Yang et al., 2016). For instance, Hazen et al. (2010) identified members of the order *Oceanospirillales* in the γ*-Proteobacteria* class as dominant in deep-sea plumes. *Oceanospirillales* were not detected in most of the plume samples in June, while *Cycloclasticus* and *Colwellia* were dominant (more than 95% of the sequences). This indicates that the DWH *Oceanospirillales* bloomed initially, followed by *Colwellia* and *Cycloclasticus* (Redmond and Valentine, 2011; Yang et al., 2016).

Construction and analysis of 16S rRNA gene clone libraries provided useful information on the structures and responses of bacterial communities during the first stage after the spill occurred. However, the 16S rRNA gene clone library technique became less prevalent after NGS was used to investigate microbial communities.

#### Gene fingerprinting techniques

Denaturing gradient gel electrophoresis (DGGE) and temperature gradient gel electrophoresis (TGGE) are two commonly used gene fingerprinting techniques that separate different DNA/RNA in the same sample via chemical or temperature gradients. Muyzer et al. (1993) first introduced DGGE into the field of microbial ecology to describe the composition of bacterial communities. Different bands on the gel indicate different bacterial phylotypes and band intensity shows the relative abundance of certain bacterial taxa. By cutting DGGE/TGGE strips, building clone libraries, and sequencing, the information related to a band can be further interpreted. Although the information provided by these two techniques are thought to be insufficient (as there are always dozens of bands for one sample) and NGS can generate more data, DGGE/TGGE techniques are still widely used to study microbial communities as they can intuitively display the differences between different samples via gel profiles within short time periods (1–2 d for an experimental cycle).

After the DWH oil spills, DGGE provided some of the first information on naturally occurring microbial communities in the sediment from shorelines along the northern coast of the Gulf of Mexico (Lisle, 2011). Some active bacterial species exhibited increases in biomass during the degradation process. Bacosa et al. (2015a) used 16S rRNA gene-based DGGE to detect microbial community dynamics and composition with the stressor of Light Louisiana Sweet (LLS) crude oil under dark and natural light conditions. The results demonstrated that different bacterial groups were involved in the degradation of n-alkane under the dark and light conditions.

Like DGGE/TGGE, TRFLP, or sometimes T-RLP is another gene fingerprinting technique. By generating a fingerprint for pattern comparison and then complementing the data with a clone library, resolving peaks using a database, and performing multivariate analysis, TRFLP can reveal the differences in unknown microbial communities in various environmental samples (Liu et al., 1997). T-RFLP was applied to investigate the composition and succession of bacterial communities after the oil spill and successfully revealed the relatively higher contribution of the bacterial group *Flavobacteria*, which is a secondary consumer of methane, oil, or cellular decay products (Redmond and Valentine, 2011). Profiles generated by TRFLP indicated that up to 56% of the total bacteria in plume samples collected in September 2010 were affiliated with *Flavobacteria*, whereas this number was only 10–30% when 16S rRNA gene-based clone library techniques were applied (Redmond and Valentine, 2011). In a recent study, the TRFLP analysis for community composition of ammonia oxidizers in salt marshes after the DWH oil spill revealed that exposure to oil, even 2 years post-spill, could contribute to subtle changes in population dynamics of bacterial communities (Bernhard et al., 2016). Although T-RFLP could provide more authentic data compared to clone libraries, it could still underestimate community diversity, especially when complex and large amounts of data were applied. In the abovementioned study (Redmond and Valentine, 2011), some terminal restriction fragments could be assigned to multiple groups. Due to the significant overlaps between the *methanotrophs, methylotrophs*, and some other γ-*proteobacteria*, it was not possible to distinguish the δ-*proteobacteria, Actinobacteria*, and SAR406 clades from each other. Nevertheless, some groups, such as *Flavobacteria*, α-*proteobacteria*, and γ-*proteobacteria* could be readily differentiated.

#### Other classic molecular methods

In addition to the aforementioned molecular methods, other molecular tools were also used to study indigenous microbial communities, such as FISH and real-time quantitative polymerase chain reaction (qPCR). The FISH method has been widely used to investigate composition of bacterial communities in recent years. It can provide fluorescent images indicating the existence and relative abundance of target genes combing with special fluorescent probes. 16S rRNA genes or functional genes can be detected by this approach without PCR amplification (DeLong et al., 1989; Amann and Fuchs, 2008). Catalyzed reporter deposition in combination with FISH (CARD–FISH) were performed to detect the microbial aggregate formations in a microcosm study using deep plume seawater with addition of nutrients (Kleindienst et al., 2015). The results revealed that the potential dispersant-degrader, *Colwellia* was one of the dominant bacterial phylotypes in microaggregate which indicated that this bacterial group could play an important role in marine oil snow formation. By developing two new 16S rRNA-targeted oligonucleotide probes (Mrb-0625-aandMrb-0625-b) for the FISH assay, McKay et al. (2016) successfully monitored the rapid increase of the oil-degrading bacterium, *Marinobacter*, after amending n-hexadecane in an enrichment experiment with a deep-sea oil plume water sample. Compared to FISH, qPCR can quantify targeted functional bacterial phyla more accurately. It provides relatively accurate data via real-time monitoring of the fluorescent signals generated by fluorescent probes (Heo et al., 2010; Yuan et al., 2010). Because probes and primes are necessary for both FISH and RT-qPCR detection, only bacterial phylotypes with probes/primers can be detected and quantified, whereas some unknown groups without specific probes/primers are difficult to be detected using these methods.

### Advanced molecular techniques

Traditional approaches applied in ecological microbiology have promoted developments in this field for decades and have provided general information on the existence (FISH), composition (Clone Library, DGGE/TGGE, FISH, and T-RTLP), and abundance (Clone Library and RT-qPCR) of microbes. Nevertheless, the information generated by these approaches is finite, and the unit cost (the cost per sequence) is relatively expensive. For instance, only dozens of DGGE bands or clones could be identified by combining the techniques of DGGE and clone libraries which could only generate limited information and hardly reflected on the real composition of bacterial communities. Furthermore, the unit cost for a single sequence can be much higher by Sanger sequencing than NGS. With the development of new approaches, such as DNA/RNA microarrays, NGS, and SIP, ecological microbiology research is faced with new opportunities. These currently prevalent techniques can generate massive data sets for detailed investigation on the composition and diversity of bacterial communities in different habitats or following particular events. Some new techniques that provide impressive information have been used in investigating the succession and function of the microbial communities after the DWH oil spill.

#### DNA microarray

DNA microarrays are collections of microscopic DNA spots, representing single target genes, that are attached to a chemical matrix and arrayed on a solid surface. By DNA-DNA or DNA-RNA hybridization, DNA microarrays can provide qualitative or quantitative measurements of microbial diversity and functional gene expression (Loy et al., 2002; Bodrossy et al., 2003). This method has been used for nearly 20 years and has experienced several generations of improvement. By hybridizing target genes with probes attached to a specific microchip, DNA microarrays can provide a deep understanding of indigenous microbial communities because of its advantages of specificity, sensitivity, quantitative analysis, and high-throughput methods (Gentry et al., 2006). Two different DNA microarray methods (PhyloChip and GeoChip) have been extensively used in the field of environmental microbiology. PhyloChip can be used to analyze the diversity of microbial communities, while the GeoChip method can be used to study the activity of functional microbes by targeting functional genes. These two different microarrays can be used together to study and compare microbial communities from different treatments or environments.

Both PhyloChip and GeoChip were applied to investigate the succession of bacterial communities and reveal the functional genes in the biodegradation of oil pollution during the DWH oil spill (Bodrossy et al., 2003; Hazen et al., 2010; Beazley et al., 2012). The PhyloChip analysis of 16S rRNA microarray conducted by Hazen et al. (2010) suggested that the oil plume significantly altered the microbial community composition and structure. In addition, the functional gene-based GeoChip microarray analysis revealed significant increases in the expression of more than 1,600 genes involved in hydrocarbon degradation (BTEX, alkane, cycloalkanes, and PAH) compared to the background non-plume samples (Hazen et al., 2010). In another study, the microbial communities in the Gulf of Mexico's coastal marshes during and after the oil spill were determined via both GeoChip and PhyloChip (Beazley et al., 2012). A total of 12,018 operation taxonomy units (OTUs) were successfully identified by PhyloChip microarray-based analysis, and the result demonstrated that bacterial phyla with potential genes for oil degradation exhibited significant increases after the oil spill. For instance, bacterial phyla affiliated to *Actinomycetaceae, Dietziaceae, Nocardioidaceae, Erythrobacteraceae*, and others, which have been shown to be capable of degrading alkanes and polycyclic aromatic hydrocarbons (PAHs), dramatically increased during the oil infiltration of sediments. In contrast, bacterial phyla of *Verrucomicrobia, Cyanobacteria*, and *Planctomycete* showed obvious decreases due to their relatively low tolerance to oil pollution. GeoChip microarray-based analysis was used to identify 16,383 unique functional genes. The results indicated that a relative abundance of functional genes involved in hydrocarbon degradation (e.g., *alkB, alkH, phaB, dsrA/B*, and others) were significantly increased between June and July in the inlet sediments (Beazley et al., 2012).

A single chip can be used to analyze thousands of genes in one assay. Therefore, microarrays are becoming one of the major culture-independent identification/quantification methods. However, the microarray technique faces the challenges of natural sequence diversity and potential cross-hybridization in complex environmental bioaerosols (Li and Huang, 2006). Lack of a probe sequence and the complexity of the gene chip's interactive hybridization have been shown to induce low specificity and poor sensitivity of experimental results (Zhou and Thompson, 2002; Gentry et al., 2006).

#### 16S rRNA gene based high-throughput sequencing

The 16S rRNA gene has been applied to study the diversity of bacterial communities via cultivation, cloning, T-RFLP, and other traditional microbiological approaches. While the information generated from these approaches can preliminarily illustrate the composition of bacterial communities, their application has been restricted by limited information, high cost, and omission of “rare biospheres.” The NGS techniques, e.g., Roche's 454 GS20 pyrosequencing (Margulies et al., 2005), Illumina Hiseq/Miseq sequencing (Bennett, 2004), ion torrent sequencing (Rothberg et al., 2011), and “single-molecule real-time” (SMRT) sequencing (Eid et al., 2009), allowed the generation of massive data sets at a much lower cost (Medini et al., 2008; Armougom and Raoult, 2009; Klindworth et al., 2013).

The DWH oil spill occurred in 2010, when the use of high-throughput sequencing was on the rise. The new techniques of microbial ecology were comprehensively applied after the event to reveal the impacts of the oil spill on bacterial communities and the responses of different bacterial phyla (Gutierrez, 2011). 454 pyrosequencing of the 16S rRNA gene was the first NGS technique applied in this event (Bælum et al., 2012), providing information on the succession of bacterial communities. The results, based on the NGS data, specifically showed the increase in the bacterial phyla *Colwelliaceae* and *Oceanospirillales*, which was consistent with the results from 16S rRNA gene-based clone library and PhyloChip (Hazen et al., 2010; Redmond and Valentine, 2011). The results were based on the analysis of 148,276 quality-filtered reads from 16 samples, whereas the first study after the spill which applied 16S rRNA gene based clone library only generated 250 high qualitied sequences from three samples (Hazen et al., 2010). The great amount of sequencing could produce more useful information for revealing the bacterial responses to the oil spill. Illumina sequencing was also used in later studies (Mason et al., 2012, 2014b; Simister et al., 2016). For instance, by analyzing massive amounts of data generated by Illumina sequencing, Simister et al. (2016) found that the dominant bacteria in both sediment and floc samples were *Proteobacteria* (55–64%). They also found that the bacterial composition of the floc samples (mostly aerobic or facultative aerobic phylotypes including *Rhizobiales, Rhodobacterales, Sphingomonadales, Rickettsiales, Alteromonadales*, and *Pseudomonadales*) was different from those in the sediment samples (aforementioned aerobic species and anaerobic phylotypes such as *Desulfobacterales, Desulfuromonadales*, and *Desulfarculales*).

#### Metagenomics

Metagenomics is a genetic analysis aimed at studying the genetic composition and community structure of microorganisms by directly analyzing DNA mixtures from microbial communities in different environmental samples (Warnecke and Hess, 2009; Coyotzi et al., 2016). Metagenomic approaches have revolutionized our ability to explore the microbial world by producing results that conventional cloning and sequencing methods have not been able to achieve (Gutierrez, 2011). 16S rRNA gene based analysis mainly characterize a bacterial community by defining its composition, succession and the relative abundance of different groups, while metagenomics analysis can afford useful metabolic information for investigating microbe functions (Scholz et al., 2012). It is helpful to fully understand uncultured microbes and provide a complete understanding of microbial activity at community levels. Specifically, metagenomic approaches enable a mechanistic understanding of the bioremediating processes and can provide some hints for optimizing the efficiency of bioremediation (Techtmann and Hazen, 2016). For instance, by analyzing metagenomic sequence data, some overlooked but important functional executor and functional genes can be explored (Jackson et al., 2015; Campeão et al., 2017).

Before NGS became prevalent, metagenomic analysis based on shotgun sequencing data was constrained by the limited data on individual genomes and environmental genetic data. The high-throughput, short runtime, and low-cost attributes of NGS have altered our overview of microbial metagenomics (Scholz et al., 2012). By applying metagenomic analysis, (Mason et al. (2012, 2014b)) investigated the responses of bacterial communities in deep sea oil plumes and sediment. Metagenomic data from these two studies demonstrated that the indigenous microbiota contributed an important ecosystem service of oil remediation in the Gulf of Mexico. Functional genes encoding for the degradation of aliphatic and simple aromatics hydrocarbon were significantly enriched after the oil spill. Nevertheless, no dramatic changes were found in the abundance of genes involved in the degradation of PAHs. This indicated that recalcitrant compounds could not be actively degraded at the sampling time and that these compounds might need a longer period for biodegradation. Another study based on metagenomic analysis found that a greater number of δ*-proteobacteria* and anaerobic functional genes were present in sediments closer to the DWH blowout site, which indicated that δ*-proteobacteria* might play a dominant role in anaerobic hydrocarbon degradation (Kimes et al., 2013).

#### Metatranscriptomics

Metagenomics can provide useful information on the genomic potential of a microbial community, whereas metatranscriptomics can be used to investigate genes that are transcribed under certain environmental conditions. It can assess microbial gene expression in a special environment or under unusual conditions via pyrosequencing of total RNA directly extracted from natural microbial assemblages (Shi et al., 2009; Warnecke and Hess, 2009). Metatranscriptomic data takes into account the dynamic state of RNA levels and thereby overcomes the constraint of metagenomics which relies on DNA for the reconstruction of metabolic models to improve prediction accuracy. Thus, metatranscriptomic analysis could provide supporting data or more convincing data in comparison to metagenomic analysis. In addition to metagenomic analysis, Mason et al. (2012) conducted metatranscriptomic analysis to determine the expressed functional genes in the active microbial community in the oil plume. Metatranscriptomic data revealed more pronounced differences in the relative abundance of active genes that have the potential for biodegradation compared to DNA-based analysis. These active genes were mostly associated with aliphatic hydrocarbon degradation but not PAH degradation, which is consistent with metagenomic analysis. In a study that considered the microbial communities of methane degradation (Lesniewski et al., 2012), data generated from metatranscriptomics revealed that the bacterial communities of both the plume and background samples were dominated by the same groups of methanotrophs and chemolithoautotrophs despite marked increases in plume total RNA concentrations (3–4 times) and microbial-mediated manganese oxidation rates (15–125 times background levels).

#### Single-cell sequencing

Single-cell sequencing is a newly developed technique to obtain genomes of individual microorganisms. This technique was first reported by Raghunathan et al. (2005) and was selected as the method of the year in 2013 by “Nature Publishing Group.” Single cells can be first isolated from various environments (soil, seawater, marine sediments, and human gastrointestinal tracts) by micro manipulation, flow cytometry or microfluidics. The whole genomes of selected single cells are then amplified by multiple displacement amplification (MDA) to generate sufficient amounts of genetic material for sequencing. Genomes can be sequenced by NGS and analyzed by new methods in bioinformatics (SPAdes) (Woyke et al., 2009). By generating genomic information on previously inaccessible species from different environments, the structure of microbial communities and the physiology of single cells can be deeply investigated. Mason et al. (2012) successfully obtained two single-cell genomes of the genus *Oceanospirillales*, which have been shown to be dominant phylotypes after the DWH oil spill (Hazen et al., 2010; Redmond and Valentine, 2011). Single-cell sequencing revealed that both cells possessed genes encoding for n-alkane and cycloalkane degradation. Furthermore, the genomic information helped in the reconstruction of the near-complete pathway for cyclohexane oxidation in the two single cells. This provided powerful evidence proving that bacteria in the order *Oceanospirillales* were responsible for the biodegradation of aliphatic hydrocarbons in the deep sea (Mason et al., 2012). Thus, with the support of metagenomic sequencing data, single-cell sequencing analysis (e.g., the genomic properties of *Colwellia*) could extend our understanding of the successional changes of the dominant microbial players (Mason et al., 2014a) and their specific metabolic activities in biochemical cycle (Musat et al., 2012).

### Bioinformatic analysis

Methods based on NGS mentioned above and DNA microarrays revolutionized microbial studies with vast quantities of data at a relatively low cost. However, the analysis of enormous amounts of data and interpretation of their biological meaning are still a great challenge. A large number of software tools (ST) and databases (DB) have been produced for bioinformatic analysis, including Greengenes (DB; DeSantis et al., 2006), Ribosomal Database Project (RDP, DB; Cole et al., 2008), SILVA (DB; Quast et al., 2012), MEGA6 (ST; Tamura et al., 2013), and QIIME (ST; Caporaso et al., 2010) for 16S rRNA gene data analysis; and Kyoto Encyclopedia of Genes and Genomes (KEGG, DB; Ogata et al., 1999), The Carbohydrate-Active EnZymes (CAZY, DB; Cantarel et al., 2008), Clusters of Orthologous Groups of proteins (COGs, DB; Tatusov et al., 2001), MetaCyc (DB; Caspi et al., 2006), and RefSeq (DB; Pruitt et al., 2005), Transporter Classification Database (TCDB, DB; Saier et al., 2016), MetaPathways (ST; Konwar et al., 2013), RAxML version 8 (ST; Stamatakis, 2014), and Circos (ST; Krzywinski et al., 2009) for analyzing metagenomics, metatranscriptomics, and single genome data. A series of visualization tools have also been developed in recent years, including Tablet (Milne et al., 2009), Integrative Genomics Viewer (IGV) (Robinson et al., 2011), Sequence Annotation, Visualization, and ANalysis Tool (Savant) Genome Browser (Fiume et al., 2010), MagicViewer (Hou et al., 2010), and Cytoscape (Kohl et al., 2011). In the microbiological studies on the DWH oil spill, these ST and databases were broadly used for analyzing DNA- and RNA-based data. Moreover, Mason et al. (2012) applied multiple ST and databases to interpret biological meaning from a mass of metagenome, metatranscriptome, and single-cell sequencing data. For instance, the GeoChip database (He et al., 2010) was applied to blast proteins involved in hydrocarbon degradation based on raw metagenomic, metatranscriptomic and single-cell reads, while the database of COGs was used to estimate genome sequence completeness. Reads from single cells were assembled using Velvet (Zerbino and Birney, 2008). Unassembled metatranscriptomic reads were mapped to the single-cell draft genome using the CLC Genomics Workbench (CLC bio). Assembled single-cell data were annotated using CAMERA (v2.0.6.2) (Seshadri et al., 2007). Clustered regularly interspaced short palindromic repeat regions were identified in the draft genome using CRISPRFinder (Grissa et al., 2007). Although bioinformatic tools have been vastly applied and greatly stimulated the data analysis, there is urgent need for the establishment of more comprehensive databases or big data sets. Meanwhile, equipment simplification, standardization methods and more user-friendly bioinformatic tools need to be developed (Capobianchi et al., 2013).

## Perspectives

### What kind of methods should be used in future studies?

**Figure 1:**
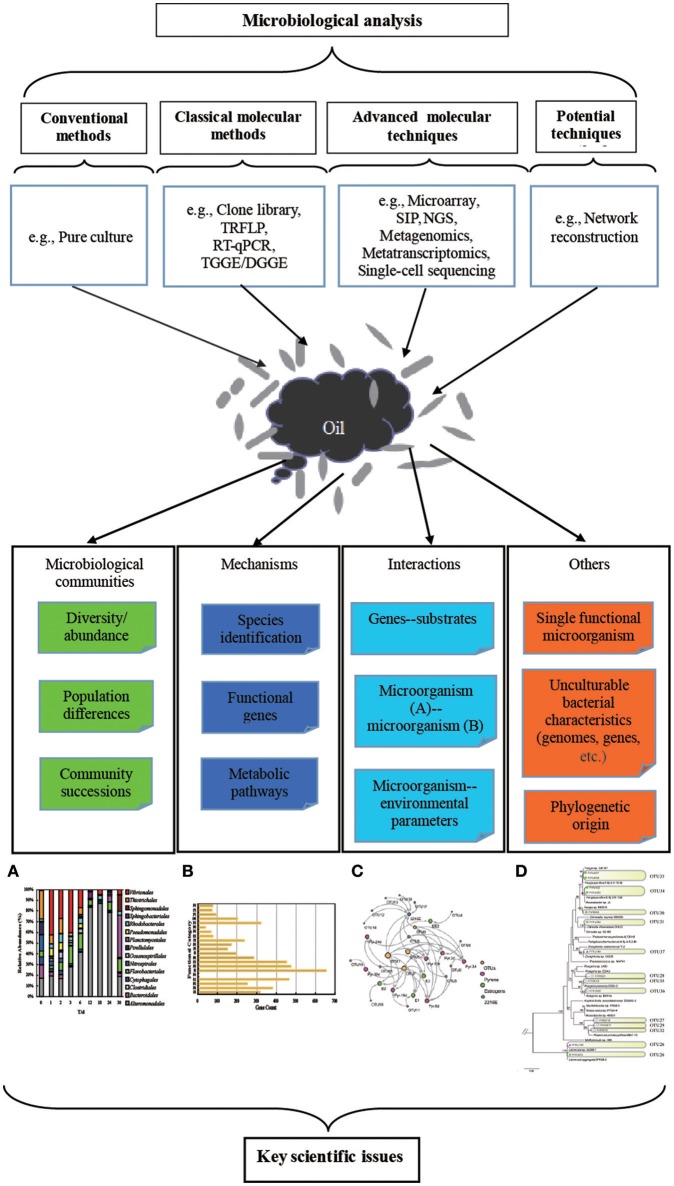
Summary of various methods that have been applied and techniques that could be applied, but have not yet been conducted, to investigate the responses of microbial communities to the DWH oil spill. **(A)** The histogram based on 16S rRNA gene-based high-throughput data can reveal microbial diversity and community succession. **(B)** The histogram based on genomic and transcriptomic data analysis can identify and classify different functional genes involved in different pathways based on the COG database. **(C)** The reconstructed molecular ecological network between microorganisms based on 16S rRNA gene, metagenomic, metatranscriptomic, and metaproteomic data can indicate the interactions and co-acclimation of microorganisms in a changing environment. **(D)** Phylogenetic relationship and community succession can be detected by generating a phylogenetic tree based on 16S rRNA gene and genomic information. All these figures were generated based on our own unpublished data.

The culture-dependent and culture-independent methods discussed in this review can partially reveal the responses of indigenous bacterial communities to the DWH oil spill, whereas a full map of bacterial community composition and activity cannot be described by a single method (Figure 1). Biodegradation efficiency and metabolite mechanisms can be adequately researched by obtaining pure cultures of bacterial strains. However, the uncultivability of bacteria from marine environments (>99% of microorganisms resist cultivation in the laboratory Kaeberlein et al., 2002) is a dramatic obstacle for revealing the real executors of *in situ* biodegradation. The 16S rRNA gene-based culture-independent methods, including DGGE, clone libraries, and NGS, can be comprehensively used to identify the bacterial composition in oil spill sites, but these methods provide limited information on metabolism of bacterial communities. Metagenomics and metatranscriptomics based on NGS can provide detailed information on a broad overview of the metabolic potentiality of bacterial communities in the polluted environment at the DNA and RNA levels, respectively. Nevertheless, these two meta-omics techniques provide limited information on the specificity of a metabolic process or the differences due to physiological or environmental conditions. The amplification bias is another obstacle in the application of these two meta-omics techniques (Lasken, 2012). Single-cell sequencing can provide genomic information on uncultivable bacteria, and it can help predict the potential pathways of biodegradation performed by the studied cell. However, single cell DNA/RNA sequences usually have high amplification bias. This is usually caused by some genomic regions being amplified more than others (Ning et al., 2014). Furthermore, analysis of single cell sequencing data always needs support from meta-omics data for additional analysis. In addition, the determination of metabolic products is also necessary for unraveling of environmental PAH biodegradation processes and also to describe microbial interactions effecting the biodegradation process (Vila et al., 2015). Therefore, a combination of different approaches is necessary for a full understanding of the composition and activity of bacterial communities. Application of integrated approaches could: (1) deeply describe the components of a complex community, including existing organisms, functions, and their activities at a given time point (Zimmerman et al., 2014); (2) identify key players, sometimes diverse, rare taxa instead of majority groups (Kleindienst et al., 2016); (3) reveal more complicated microbial interactions by determining the connections of both cultured and currently uncultured microorganisms (Abraham, 2014); (4) predict functions of the whole community with the help of different bioinformatic tools which can improve prevention and remediation of potential perturbations (Ivshina et al., 2015).

In the first microbial study after the oil spill, Mason et al. (2010) applied the PhyloChip 16S ribosomal RNA (rRNA) microarray and clone library to study the bacterial community composition in the oil plume. The authors successfully isolated oil-degrading bacteria by a convenient cultivation method. Additionally, multivariate analysis of phospholipid fatty acid (PLFA) was applied in this study to analyze the composition of bacterial communities by identifying different assemblages of PLFA. Mason et al. (2012) investigated the role of the indigenous microbial community by combining different molecular methods including 16S rRNA gene sequencing, metagenomics, metatranscriptomics, and single-cell genomics. These methods based on NGS are currently prevalent in the field of microbial ecology, and they can provide abundant information for thorough analysis of the composition and behavior of the bacterial communities.

Analysis based on SIP is a typical strategy for bacterial community study that combines culture-dependent and culture-independent approaches. First, cultivation is necessary to obtain molecular markers (DNA, RNA, proteins, or PLFA) that are labeled with a stable isotopic element (e.g., ^13^C, ^15^N, and ^18^O). The labeled molecular markers can then be analyzed by 16S rRNA gene sequencing (clone library or NGS), metagenomic analysis, metatranscriptomic analysis, and mass spectrometer (MS) analysis. These methods can be used to generate data to reveal the bacterial community and activity. SIP analysis is a powerful tool for linking microbial species and metabolism (Dumont and Murrell, 2005). By using DNA-SIP in combination with 16S rRNA gene clone libraries and T-RFLP analysis, Redmond and Valentine (2011) demonstrated that bacteria in the genus *Colwellia* were active in ethane and propane oxidation in oil polluted deep-sea water plumes. Mason et al. (2014b) used surface sediments polluted by the DWH oil spill and ^14^C-labeled hydrocarbons to perform mineralization experiments. They successfully found that the key hydrocarbon degradation pathway by sediment microbes was as follows: propylene glycol, dodecane, toluene, and phenanthrene. By monitoring the biodegradation efficiency of ^13^C-labeled different hydrocarbons (aliphatic and aromatic), Gutierrez et al. (2013) applied 16S rRNA gene-based NGS and qPCR detection to reveal the activity of bacterial communities. The results indicated that the different bacterial phyla were responsible for the degradation of different hydrocarbons. For instance, bacteria in the genera *Alcanivorax* and *Marinobacter* were found to be enriched in the experimental setups with the addition of aliphatic hydrocarbons, whereas *Alteromonas, Cycloclasticus*, and *Colwellia* dominated the experimental setups supplemented with polycyclic aromatic hydrocarbon.

### How to illustrate the cooperation of different bacteria?

A global perspective calls for a deep understanding of the composition and activity of the bacterial communities in a relevant environmental context (Faust and Raes, 2012). We should not only focus on functional bacterial communities that are directly degrading pollutants, but also on the other biological components involved in the biodegradation or bioremediation processes via chemical and biological interactions (Head et al., 2006). For instance, microorganisms without oil-degrading capability could also be involved in the biodegradation process by consuming the metabolites released by oil-degrading bacteria. Some non-oil-degrading microorganisms can either provide limiting nutrients or produce surfactant or emulsification of oil and water for the primary oil degraders (Head et al., 2006). Reconstructing the microbial network might be an efficient method for investigating the complicated interactions among indigenous bacteria. Zhou et al. (2010; 2011) developed a random matrix theory (RMT)-based conceptual framework to reconstruct functional molecular ecological networks based on metagenomics and genechip data. This approach was applied to evaluate the responses of bacterial communities under long-term, free-air CO_2_ enrichment (Zhou et al., 2010, 2011). The reconstructed networks revealed dramatic changes in the interactions among different phylogenetic groups/populations. The changes in network structure were significantly correlated with nutrient contents. In our previous study, we investigated the responses and activities of bacterial communities under stresses from hydrocarbons with different structures by reconstructing microbial networks and predicting the individual roles of functional bacteria in the key nodes of the networks. The interactions of different bacteria in the networks helped microbes co-acclimate to the changing environment and initiate biodegradation (Wang et al., 2016). In all, the microbial networks can provide additional information besides the composition of bacterial communities. Most importantly, the key functional bacterial phyla, including the degrading conductor and tightly associated non-degrading bacteria, could be identified (key nodes in the network). Based on this information, the bacterial communities in polluted sites can be manipulated for enhancing the abundances of beneficial species and functions for bioremediation (Faust and Raes, 2012). The key strategies for applications based on network reconstruction and analysis is engineering a whole community instead of a single species (Curtis et al., 2003; Dunham, 2007).

## Conclusions

The impacts of the DWH oil spill are gradually fading, hence the research on its impacts on indigenous microbial communities is decreasing. However, current literatures adequately address the approaches for investigating the composition and activity of microbial communities. In this review, we summarized various culture-dependent and culture-independent techniques applied in the study of microbes after the DWH oil spill. Conventional methods, such as pure culture and classical molecular biological methods (e.g., Clone Library, TRFLP, TGGE/DGGE, and RT-qPCR), provide qualitative impressions about the functional bacteria conducting biodegradation. Prevalent microbiological molecular techniques (e.g., microarrays, NGS, metagenomics, metatranscriptomics, and single-cell sequencing) have also generated a great amount data on bacterial composition and predicted potential pathways for hydrocarbon degradation. For future investigation, we recommend an informed application of prevalent NGS-based genomic technologies and microbial network reconstruction in conjunction with conventional culture-dependent approaches. Especially, integrated application of different approaches could afford a more comprehensive view of indigenous bacterial communities and their potential functions in polluted sites.

## Author contributions

SZ, ZH, and HW provided substantial contributions to the conception and design of the work. SZ and HW drafted the work. SZ, ZH and HW agree to be accountable for all aspects of the work in ensuring that questions related to the accuracy or integrity of any part of the work are appropriately investigated and resolved.

### Conflict of interest statement

The authors declare that the research was conducted in the absence of any commercial or financial relationships that could be construed as a potential conflict of interest.
